# TRK Protein Expression in Merkel Cell Carcinoma Is Not Caused by *NTRK* Fusions

**DOI:** 10.3390/ijms232315366

**Published:** 2022-12-06

**Authors:** Rocco Cappellesso, Lorenzo Nicolè, Paolo Del Fiore, Luisa Barzon, Alessandro Sinigaglia, Silvia Riccetti, Renato Franco, Federica Zito Marino, Giada Munari, Carolina Zamuner, Francesco Cavallin, Marta Sbaraglia, Francesca Galuppini, Franco Bassetto, Mauro Alaibac, Vanna Chiarion-Sileni, Luisa Piccin, Clara Benna, Matteo Fassan, Simone Mocellin, Angelo Paolo Dei Tos

**Affiliations:** 1 Pathological Anatomy Unit, Padua University Hospital, 35121 Padua, Italy; 2 Department of Pathology, Angelo Hospital, 30174 Venice, Italy; 3 Department of Medicine (DIMED), University of Padua, 35121 Padua, Italy; 4 Soft-Tissue, Peritoneum and Melanoma Surgical Oncology Unit, Veneto Institute of Oncology IOV-IRCCS, 35128 Padua, Italy; 5 Department of Molecular Medicine (DMM), University of Padua, 35121 Padua, Italy; 6 Pathology Unit, University of Campania “L. Vanvitelli”, 80129 Naples, Italy; 7 Veneto Institute of Oncology IOV-IRCCS, 35128 Padua, Italy; 8 Independent Statistician, 36020 Solagna, Italy; 9 Department of Neurosciences (DNS), University of Padua, 35128 Padua, Italy; 10 Melanoma Unit, Oncology 2 Unit, Veneto Institute of Oncology IOV-IRCCS, 35128 Padua, Italy; 11 Department of Surgery, Oncology and Gastroenterology (DISCOG), University of Padua, 35128 Padua, Italy

**Keywords:** *NTRK1*, *NTRK2*, *NTRK3*, Merkel cell carcinoma, EPR17341

## Abstract

Merkel cell carcinoma (MCC) is a rare and aggressive cutaneous malignant tumor with neuroendocrine differentiation, with a rapidly growing incidence rate, high risk of recurrence, and aggressive behavior. The available therapeutic options for advanced disease are limited and there is a pressing need for new treatments. Tumors harboring fusions involving one of the neurotrophin receptor tyrosine kinase (*NTRK*) genes are now actionable with targeted inhibitors. *NTRK*-fused genes have been identified in neuroendocrine tumors of other sites; thus, a series of 76 MCCs were firstly analyzed with pan-TRK immunohistochemistry and the positive ones with real-time RT-PCR, RNA-based NGS, and FISH to detect the eventual underlying gene fusion. Despite 34 MCCs showing pan-TRK expression, *NTRK* fusions were not found in any cases. As in other tumors with neural differentiation, TRK expression seems to be physiological and not caused by gene fusions.

## 1. Introduction

Merkel cell carcinoma (MCC) is a rare primary malignant tumor of the skin with neuroendocrine differentiation [1,2,3]. The reported incidence of MCC has increased in recent years and varies across the world, ranging from 0.3 to 1.6 per 100,000 people per year depending on the distribution of the two etiologic factors, namely, ultra-violet (UV) radiation exposure and Merkel cell polyomavirus (MCPyV) infection [4,5,6,7]. MCC is usually diagnosed in the seventh decade of age in advanced stage of disease and features a highly aggressive and rapid course with an overall 5-year survival rate of about 40% in some series [4,7,8,9]. MCPyV-negative MCC is associated with a higher number of molecular alterations and a worse prognosis than its MCPyV-positive counterpart [3]. MCC systemic treatment was based on platinum agents and etoposide chemotherapy until recently, when the paradigm shifted to immune checkpoint inhibitors that were demonstrated to be much more effective in attaining a durable response in many cases [1,8,10,11,12]; however, approximatively half the patients were unresponsive, thus underscoring a critical need to find novel treatments [1].

Neurotrophin receptor tyrosine kinase genes *NTRK1*, *NTRK2*, and *NTRK3,* respectively, encode for cell-surface receptor tyrosine kinase proteins TRKA, TRKB, and TRKC (collectively named as TRK proteins) [13]. These receptors are physiologically expressed in the neural tissue and can be activated by binding with various ligands, such as nerve-growth factor (NGF), brain-derived neurotrophic factor (BDNF), and neurotrophin-3 and -4 (NT-3 and -4) [13]. TRK activation triggers the autophosphorylation of the intracellular tyrosine residues and, thus, the transmission of the signal through several pathways that ultimately regulates the transcription of genes involved in neuronal survival and differentiation [13]. Chromosomal translocations involving one of the *NTRK* genes may result in fused genes that produce TRK chimeric proteins with oncogenic properties when combining constitutive expression with ligand-independent activation. These alterations occur across a wide range of adult and pediatric tumors and are recurrent in some specific histotypes (salivary gland and breast secretory carcinoma, mesoblastic nephroma, and infantile fibrosarcoma) and are very infrequent in several other more common tumors [13,14,15,16,17,18,19,20]. Importantly, *NTRK* fusions are clinically actionable; indeed, the TRK inhibitors Larotrectinib and Entrectinib can achieve histology-agnostic responses in patients with *NTRK*-fused tumors [21,22]. As in the normal counterpart, TRK proteins are commonly expressed in malignant tumors with neural differentiation in the absence of *NTRK* gene family alterations [23,24]; however, *NTRK* fusions have been detected with a fair frequency in neuroendocrine malignancies of the lung, pancreas, and uterus [25,26,27,28]. No data are available about *NTRK* alterations in the neuroendocrine tumors of the skin, although a huge number of different neoplasms have already been tested for and the molecular landscape of MCC has been deeply investigated [8,20].

The aims of this study were to evaluate the expression of TRK proteins in a series of MCCs, to assess the eventual correlation with the presence of *NTRK*-fusions, and to verify the association with clinicopathological features and survival.

## 2. Results

### 2.1. Pan-TRK Immunohistochemistry

**Figure 1:**
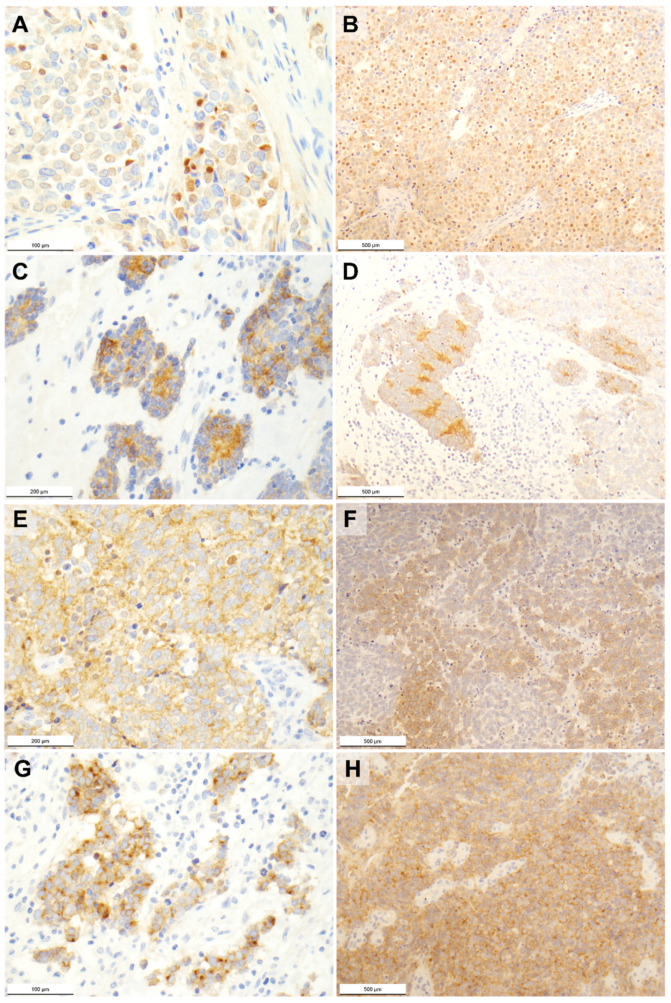
Photomicrographs of pan-TRK immunostained Merkel cell carcinomas showing (**A**,**B**) nuclear, (**C**,**D**) cytoplasmic, (**E**,**F**) membranous, and (**G**,**H**) dot-like patterns of expression. Original magnification 40× (**B**,**D**,**F**,**H**), 100× (**C**,**E**), and 200× (**A**,**G**).

Overall, 34 MCC cases (45%) showed a positive pan-TRK immunoreaction. The intensity of the immunostaining was weak in 18 tumors (53%), moderate in 12 tumors (35%), and strong in 4 tumors (12%). As for the cellular pattern of TRK expression, this regarded a single localization in 11 MCC cases and multiple localizations in the remaining ones. Pan-TRK immunostaining (Figure 1) was nuclear in 6 tumors (in 3 as single cellular compartment), cytoplasmic in 29 tumors (in 6 as single cellular compartment), membranous in 19 tumors (in 1 as single cellular compartment), and dot-like in 10 tumors (in 1 as single cellular compartment).

### 2.2. NTRK Fusion Analyses

**Figure 2:**
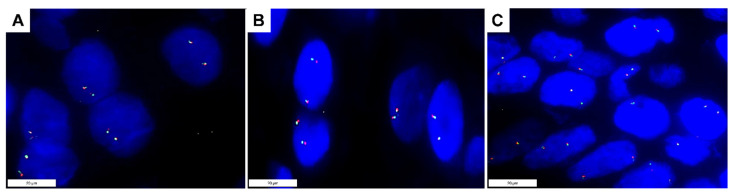
Photomicrographs of break-apart FISH analyses in a Merkel cell carcinoma with positive nuclear pan-TRK immunoreaction showing a wild type signal configuration, namely, the presence of two pairs of closely approximated or fused signals in each nucleus (ZytoLight SPEC NTRK1 (**A**), NTRK2 (**B**), and NTRK3 (**C**) Dual Color Break Apart Probe). Original magnification 1000× (**A**–**C**).

Neither real-time RT-PCR, nor RNA-based NGS, nor FISH (Figure 2) detected fusions involving *NTRK1*, *NTRK2*, or *NTRK3* in the 76 MCC cases—not even in those with positive pan-TRK immunoreaction.

### 2.3. TRK Expression and Clinicopathological Features

Overall, TRK expression was not associated with age, gender, primary site, or MCPyV infection (Table S1); even the cytoplasmic, membranous, and dot-like TRK expression patterns were not related to age, gender, primary site, or MCPyV positivity. Nuclear pan-TRK immunostaining was associated with MCPyV negativity (*p* = 0.02) but not with age, gender, and primary site (Table S1).

### 2.4. TRK Expression and Survival

Median overall survival was 31 months (IQR 14–59). At the analysis, 32 patients had tumor recurrence, clinical upstaging, or disease progression. Three-year recurrence/progression-free survival (R/PFS) was 52%, while 3-year overall survival (OS) was 53%. R/PFS and OS were not associated with overall, nuclear, cytoplasmic, membranous, or dot-like TRK expression (Table S2).

## 3. Discussion

MCC is a rare cutaneous malignant tumor with neuroendocrine differentiation, increasing incidence, high risk of recurrence, and aggressive behavior [1,2,3,29,30]. Patients with localized MCC are treated with surgery followed by adjuvant radiotherapy and, usually, show low rates of tumor relapse and mortality [12,31]. Those with metastatic MCC, instead, are treated with immunotherapy, but about half of them are unresponsive and still do not have an effective alternative treatment [1,8,10,11,12,32].

The TRK inhibitors Larotrectinib and Entrectinib have shown dramatic response in patients harboring *NTRK*-fused tumors regardless of the histotype, and a search for actionable tumors has begun [21]. Tumors with neuroendocrine differentiation belong to the group of relatively common tumors that occasionally harbor *NTRK* fusions [25,26,27,28]; indeed, *NTRK* fusions have been reported in 10 cases out of 7,424 neuroendocrine neoplasms (0.13% of frequency) [25,26,27,28]—two cases were lung adenocarcinomas with neuroendocrine features, three cases were lung large-cell neuroendocrine carcinoma, two cases were pancreatic neuroendocrine tumors, one case was a uterine neuroendocrine tumor, and the last two cases were neuroendocrine tumors of unknown primary origin [25,26,27,28]. Fusions involved *NTRK1*, *NTRK2*, and *NTRK3* genes with several mechanisms and partners: *TPR-NTRK1*, *NTRK1-CCDC19*, *NTRK1-GPATCH4*, *PIP5K1A-NTRK1*, *RFWD2-NTRK1*, *NOTCH2–NTRK1*, *SQSTM1-NTRK2*, *SQSTM1-NTRK3*, *ETV6-NTRK3*, and *NTRK3*-intergenic region [25,26,27,28]. This reflects the significant variability of *NTRK* fusion partners identified so far with the involvement of different breakpoints and exons [23,33]. Despite not all having been demonstrated to be driver alterations, i.e., critical for tumor growth and progression, it is unlikely that they are only passenger aberrations; indeed, *NTRK* fusions are usually detected in a mutually exclusive manner with the most common oncogenic drivers [20,33,34,35,36]. Considering the neuroendocrine nature of MCC, the working hypothesis of this study was that TRK expression in this tumor could be due to *NTRK* fusions in a small portion of cases.

**Figure 3:**
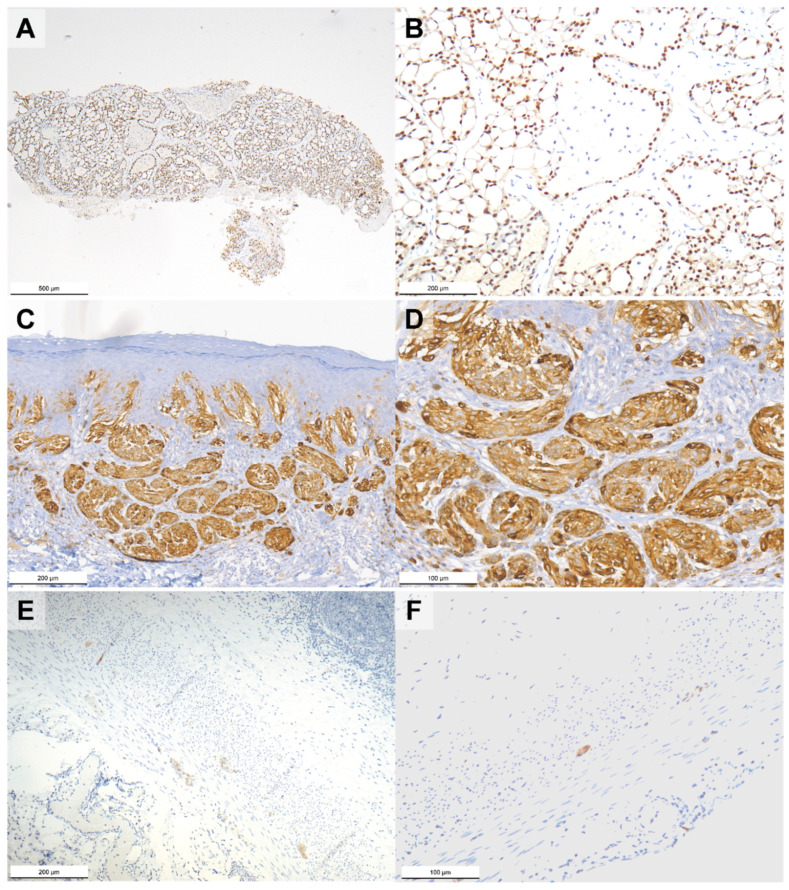
Photomicrographs of pan-TRK immunostained breast secretory carcinoma with known *ETV6-NTRK3* fusion (**A**,**B**) showing nuclear immunoreaction, atypical Spitz tumor with known *LMNA-NTRK1* fusion (**C**,**D**) showing cytoplasmic immunoreaction, and normal vermiform appendix without *NTRK1*, *NTRK2*, and *NTRK3* fusions (**E**,**F**) showing cytoplasmic immunoreaction in the ganglion cells within the muscular layer. Original magnification 40× (**A**), 100× (**B**,**C**,**E**), and 200× (**D**,**F**).

The 76 MCC cases were screened using pan-TRK immunohistochemistry, a practical and effective approach to identify TRK expressing tumors [24,33]. About half the cases showed TRK expression with variable intensity. In line with the literature, the most common staining pattern was cytoplasmic, followed by the membranous, dot-like, and nuclear cellular localizations [20,24]. Despite the fact that the antibody used was not able to distinguish between wild type and chimeric oncogenetic proteins, the subcellular staining patterns have been associated with some degree of specificity with certain fusion partners, such as nuclear positivity and *ETV6-NTRK3* fusion (Figure 3) [24]; nevertheless, tumors without *NTRK* fusions showing weak or focal pan-TRK immunostaining, even in the nuclei, have been reported [20,24,37,38].

To verify the presence of underlying *NTRK* fusions, the pan-TRK positive MCCs were tested using three different molecular techniques (RNA-based NGS, real-time RT-PCR, and FISH) to address the shortcomings of each method and to achieve solid and reliable results using archival formalin-fixed and paraffin-embedded (FFPE) samples [39]. None of the MCC cases harbored a *NTRK* fusion. This eventuality was expected considering the rarity of NTRK fusions in non-cutaneous neuroendocrine neoplasms and the physiological expression of TRK in tissues with neural differentiation and in their neoplastic counterparts [20,24]. Merkel cells are thought to be at the origin of MCC because they share many morphological and immunohistochemical hallmarks, mainly neuroendocrine features [2]. These cells are present in the basal layer of the epidermis, especially around hair follicles, are associated with afferent sensory nerves, and function as mechanoreceptors for gentle touch stimulation [2,40]. Neurotrophin signaling seems to play a key role in recruiting afferents to Merkel cell-enriched skin areas and in the development and maintenance of touch receptors [40,41]. In particular, innervation depends on the expression of TRKA during the initial development and on TRKC throughout the development of the Merkel endings [41]; thus, the present findings support that physiological expression of TRK is conserved also in some MCCs.

TRK expression is not involved in MCC carcinogenesis and tumor progression since it was not associated with prognostic and clinicopathological features, except for the nuclear localization of the receptor in MCPyV-negative tumors. MCPyV-negative cases belong to the group of MCC with the greatest amount of molecular alteration [2,3]; indeed, MCPyV-negative MCC has been associated to the UV radiation mutational signature, namely, a predominance of cytosine to thymidine transition at DNA dipyrimidine sites, and to a tumor mutational burden 25–90-fold higher than the MCPyV-positive counterpart [2,42,43,44]; thus, the nuclear TRK expression observed in some MCC cases may be due to an aberrant protein product of mutated *NTRK* genes. Another possible explanation could be that TRK is wild-type and the incorrect localization depends on alterations in other pathways that cause the internalization and nuclear migration of the receptor. These hypotheses deserve to be investigated in cell lines of Merkel cell carcinoma since no data are available on this issue.

The main strengths of the present study are the collection and analysis of a quite large series of this rare tumor and the application of different molecular techniques to overcome the limitations of each method. Nevertheless, the number of cases may be insufficient to completely rule out the presence of *NTRK* fusions in MCC, considering that in the neuroendocrine setting these have been reported in less than 0.1% of neoplasms. Future larger multicentric studies should explore the gene-fusion landscape of MCC since data are lacking in the literature.

## 4. Materials and Methods

### 4.1. Samples

This retrospective study was conducted on the archival FFPE tumor samples of 76 consecutive patients who had been diagnosed with MCC during the period 2001–2019 at the Padua University Hospital or at the Veneto Institute of Oncology. Clinicopathological features of the patients are summarized in Table 1. All cases were reviewed and the diagnoses confirmed in all instances by a pathologist, according to the fourth edition of the World Health Organization classification of skin tumors [45].

### 4.2. Immunohistochemistry

Immunohistochemistry was performed on 4 μm-thick sections from the most representative FFPE sample of each case with the Ventana pan-TRK Assay (antibody clone EPR17341; Abcam, Cambridge, MA, USA) on the automated immunostainer platform Ventana Benchmark XT (Ventana Medical Systems, Tucson, AZ, USA). Sections were then slightly counterstained with hematoxylin. Appropriate positive and negative controls were run simultaneously, as explained elsewhere [39]. Staining intensity and pattern (i.e., nuclear, cytoplasmic, membranous, or dot-like) were assessed in each case. 

### 4.3. Microdissection

Ten consecutive 10 μm-thick sections were cut from each FFPE sample using a new microtome blade to avoid cross-contamination. A final 5 μm-thick section was cut and stained with hematoxylin and eosin to confirm the presence of residual tumor. Tumor cells were then manually microdissected using sterile needles under direct microscopic visualization to ensure a tumor cell content >80% and collected in two 1.5 mL tubes, one for DNA extraction and one for RNA extraction.

### 4.4. DNA Extraction

Microdissected FFPE tumor samples were deparaffinized in xylene for 3 min at 50 °C, before nucleic acid purification. Total nucleic acids were purified with a MagNA Pure 96 nucleic acid kit in a MagNA Pure 96 system (Roche Diagnostics, Mannheim, Germany) and eluted in 100 μL, according to the manufacturer’s recommendations. Quantity and integrity of purified DNA was checked by quantitative real-time PCR amplification of the β-globin gene, which also allowed estimating the number of cells present in each sample.

### 4.5. Detection of Merkel Cell Polyomavirus DNA

About 100 ng of total DNA was used for detection of MCPyV DNA by real-time PCR assays using oligonucleotide primers and TaqMan probes, as previously reported [46]. Real-time PCR analyses were run of ABI PRISM 7900 HT Sequence Detection System (Thermo Fisher Scientific, Waltham, MA, USA). The plasmid pMCV-R17a (Addgene, Cambridge, MA) containing the complete genome of MCPyV was used as a positive control [47]. DNA extraction, water, and buffer PCR controls were used to exclude contamination, and these were consistently negative.

### 4.6. RNA Extraction

Total RNA was extracted using the RecoverAll Total Nucleic Acid Isolation Kit for FFPE (ThermoFisher Scientific) and, finally, eluted in 50 μL of DEPC-treated water. RNA concentration of 1 μL of each sample was assessed using the Qubit RNA HS Assay Kit (ThermoFisher Scientific) on a Qubit 3.0 Fluorometer (ThermoFisher Scientific). All samples were stored at −80 °C until further use.

### 4.7. Real-Time RT-PCR

Gene-fusion analysis of the *NTRK* gene family was performed with one-step real-time RT-PCR technique using the Easy PGX ready NTRK fusion kit (Diatech Pharmacogenetics, Ancona, Italy) on the EasyPGX qPCR instrument 96 (Diatech Pharmacogenetics), following the manufacturer’s instructions. This test can detect 32 different gene-fusion variants involving *NTRK1*, *NTRK2*, and *NTRK3* (detailed list in Table S3). Positive (EasyPGX NTRK Fusion positive control) and negative (water) controls were run concurrently. Data analysis was automatically performed with the EasyPGX analysis software (Diatech Pharmacogenetics; version 4.0.0).

### 4.8. RNA-Based NGS

The presence of fusions involving *NTRK1*, *NTRK2*, and *NTRK3* was also assessed in each RNA extracts using the Archer FusionPlex Oncology Research kit (ArcherDX, Boulder, CO, USA) based on the targeted enrichment method called anchored multiplex PCR (AMP). Briefly, 200 ng of total RNA was reverse transcribed to single strand cDNA using random primers and RNA quality was assessed using the Archer PreSeq RNA QC assay (ArcherDX). Samples with adequate RNA quality have been used to create the libraries, according to the manufacturer’s instructions. Libraries were quantified using the KAPA Library Quantification kit (Kapa Biosystems, London, UK) and pooled to equimolar concentration. NGS analysis was performed on a NextSeq-500 Platform (Illumina, San Diego, USA) and results were analyzed using the Archer analysis software (ArcherDX; version 6.0.4). Fusions categorized as strong by the software were required to consider a sample to be positive.

### 4.9. Fluorescence In Situ Hybridization

FISH analyses were performed on three consecutive 4 μm-thick sections cut from each FFPE sample using the BOND FISH kit (Leica Biosystems, Newcastle upon Tyne, UK) on the automated BOND system (Leica Biosystems), according to the manufacturer’s instructions. NTRK1, NTRK2, and NTRK3 genes were examined by three separate assays using specific break-apart probes for each gene: ZytoLight SPEC NTRK1 Dual Color Break Apart Probe (ZytoVision, Bremerhaven, Germany), ZytoLight SPEC NTRK2 Dual Color Break Apart Probe (ZytoVision Bremerhaven), and ZytoLight SPEC NTRK3 Dual Color Break Apart Probe (ZytoVision Bremerhaven). Slides were counterstained with 4′,6-diamidine-2′-phenylindole dihydrochloride (DAPI) in antifade solution and evaluated using an automated CytoVision platform (Leica Biosystems). FISH interpretation was performed with the automated fluorescence microscope Leica DM5500 B (Leica Biosystems) using the filter ET-D/O/G for double Spectrum Green plus Spectrum Orange. FISH signals were counted in at least 50 non-overlapping intact nuclei. A classic break-apart pattern with one fusion signal and two separated orange and green signals or an atypical pattern with one fusion signal and a single orange signal without a corresponding green signal was required to consider the assay positive (regardless the percentage of nuclei showing one of these two signal patterns).

### 4.10. Statistical Analysis

Continuous data were expressed as median and interquartile range (IQR), and categorical data as frequency and percentage. The association between clinicopathological features was investigated using Fisher’s exact test or the Mann–Whitney test. Survival curves were calculated using the Kaplan–Meier method. The association between variables of interest and survival (R/PFS and OS) was evaluated using Cox regression models, with effects sizes expressed as hazard ratio with 95% confidence interval (CI). All tests were two-sided and a *p* < 0.05 was considered statistically significant. Statistical analysis was performed using R 4.0 (R Foundation for Statistical Computing, Vienna, Austria) [48].

## Figures and Tables

**Table 1:** Clinicopathological characteristics of patients included in the study (n = 76).

Category	n	%
Age at diagnosis (yrs)		
- Mean (SD) - Range	72.2 (11.5) 45–95	
Gender		
- Male - Female	36 40	47 53
Primary site		
- Eyelid - Head and neck - Trunk - Extremity	3 20 8 45	4 26 11 59
MCPyV		
- Positive - Negative	43 33	57 43

MCPyV = Merkel cell polyomavirus; SD = standard deviation.

## Supplementary Materials

The following supporting information can be downloaded at: https://www.mdpi.com/article/10.3390/ijms232315366/s1.
