# Supplementary material for: TRK Protein Expression in Merkel Cell Carcinoma Is Not Caused by NTRK Fusions
Source: Int J Mol Sci. 2022 Dec 6;23(23):15366. doi: 10.3390/ijms232315366 (PMC9737899; doi:10.3390/ijms232315366)
Supplement: Supplementary file 1 [file ijms-23-15366-s001.zip › ijms-2039232-supplementary.pdf]

International Journal of
Molecular Sciences

MDPI

Table S1. Association between TRK expression and clinicopathological features.

Comparison	Variable	Groups		p-value
**Pan-TRK expression**		**Negative pan-TRK**	**Positive pan-TRK**	
	N	42	34	-
	Age, years: median (IQR)	75 (68-83)	69 (64-78)	0.06
	Males: n (%)	17 (40)	19 (56)	0.29
	Primary site, n (%):<br>Extremities<br>Eyelid<br>HN<br>Trunk	 21 (50)<br>3 (7)<br>13 (31)<br>5 (12)	 24 (71)<br>0<br>7 (20)<br>3 (9)	0.22
	MCPyV positive: n (%)	26 (62)	17 (50)	0.42
**Membranous TRK expression pattern**		**Not-membranous TRK expression**	**Membranous TRK expression pattern**	
	N	15	19	-
	Age, years: median (IQR)	67 (62-77)	70 (65-81)	0.37
	Males: n (%)	9 (60)	10 (53)	0.74
	Primary site, n (%):<br>Extremities<br>Eyelid<br>HN<br>Trunk	 10 (67)<br>2 (13)<br>3 (20)	 14 (74)<br>5 (26)<br>0 (0)	0.11
	MCPyV positive: n (%)	8 (53)	9 (47)	0.99
**Cytoplasmic TRK expression pattern**		**Not-cytoplasmic TRK expression**	**Cytoplasmic TRK expression**	
	N	5	29	-
	Age, years: median (IQR)	66 (63-69)	70 (65-78)	0.73
	Males: n (%)	3 (60)	16 (55)	0.99
	Primary site, n (%):<br>Extremities<br>Eyelid<br>HN<br>Trunk	 4 (80)<br>1 (20)<br>0 (0)	 20 (69)<br>6 (21)<br>3 (10)	0.99
	MCPyV positive: n (%)	1 (20)	16 (55)	0.34
**Dot-like TRK expression pattern**		**Not dot-like TRK expression**	**Dot-like TRK expression**	
	N	23	10	-
	Age, years: median (IQR)	67 (62-79)	72 (66-77)	0.38
	Males: n (%)	14 (61)	4 (40)	0.45
	Primary site, n (%):<br>Extremities<br>Eyelid<br>HN<br>Trunk	 15 (65)<br>6 (26)<br>2 (9)	 8 (80)<br>1 (10)<br>1 (10)	0.72
	MCPyV positive: n (%)	13 (57)	4 (40)	0.46
**Nuclear TRK expression pattern**		**Not-nuclear TRK expression**	**Nuclear TRK expression**	
	N	28	6	-
	Age, years: median (IQR)	69 (62-78)	68 (66-74)	0.79

	Males: n (%)	15 (54)	4 (67)	0.67
	Primary site, n (%):<br>Extremities<br>Eyelid<br>HN<br>Trunk	 20 (72)<br>6 (21)<br>2 (7)	 4 (66)<br>1 (17)<br>1 (17)	0.78
	MCPyV positive: n (%)	17 (61)	0 (0)	**0.02**

IQR: interquartile range. Information on dot-like TRK expression pattern was not available in one patient.

Table S2. Analysis of survival.

**Variable**	**Recurrence/progression-free survival**		**Overall survival**	
	**Hazard ratio (95% confidence interval)**	**p-value**	**Hazard ratio (95% confidence interval)**	**p-value**
Negative pan-TRK<br>Positive pan-TRK	Reference<br>1.39 (0.69 to 2.77)	0.36	Reference<br>1.01 (0.56 to 1.84)	0.97
Not-membranous TRK expression<br>Membranous TRK expression	Reference<br>1.03 (0.38 to 2.76)	0.96	Reference<br>1.02 (0.41 to 2.53)	0.97
Not-cytoplasmic TRK expression<br>Cytoplasmic TRK expression	Reference<br>0.58 (0.16 to 2.039	0.39	Reference<br>0.61 (0.20 to 1.87)	0.38
Not dot-like TRK expression<br>Dot-like TRK expression	Reference<br>0.76 (0.24 to 2.40)	0.64	Reference<br>1.41 (0.51 to 3.90)	0.51
Not-nuclear TRK expression<br>Nuclear TRK expression	Reference<br>0.34 (0.05 to 2.60)	0.30	Reference<br>2.14 (0.76 to 6.03)	0.15

Table S3. List of detectable gene alterations with the Easy PGX ready NTRK fusion test.

NTRK1 exon 9-10	*TFG-NTRK1* T5:N9<br>*NFASC-NTRK1* N20:N10<br>*NFASC-NTRK1* N21:N10
NTRK1 exon 10	*SQSTM1-NTRK1* S4:N10<br>*TFG-NTRK1* T5:N10<br>*TPM3-NTRK1* T7:N10<br>*TPR-NTRK1* T21:N10<br>*TRIM63-NTRK1* T8:N10
NTRK1 exon 11-12del	*BCAN-NTRK1* B13:N11<br>*LMNA-NTRK1* L11del178:N11<br>*LMNA-NTRK1* L2:N11<br>*PPL-NTRK1* P22del2181:N11<br>*TPR-NTRK1* T6del122:N12del99
NTRK1 exon 12	*MPRIP-NTRK1* M21:N12<br>*MPRIP-NTRK1* M18:N12<br>*MPRIP-NTRK1* M14:N12<br>*SCYL3-NTRK1* S11:N12<br>*TPM3-NTRK1* T7:N12<br>*TPR-NTRK1* T6:N12
NTRK2 exon 12-15	*AFAP1-NTRK2* A13:N12<br>*VCL-NTRK2* V16:N12<br>*TLE4-NTRK2* T7:N15<br>*TRIM24-NTRK2* T12:N15
NTRK2 exon 16-17	*AGBL4-NTRK2* A6:N16<br>*QKI-NTRK2* Q6:N16<br>*SQSTM1-NTRK2* S4:N16<br>*STRN-NTRK2* S3:N16<br>*SQSTM1-NTRK2* S4:N17
NTRK3 exon 14	*ETV6-NTRK3* E4:N14<br>*ETV6-NTRK3* E5:N14
NTRK3 exon 15	*ETV6-NTRK3* E4:N15<br>*ETV6-NTRK3* E5:N15
